# Predictors of Successful Extubation in Reintubated Patients: The Impact of Fluid Balance during the 24 Hours Prior to Extubation

**DOI:** 10.5005/jp-journals-10071-23212

**Published:** 2019-07

**Authors:** Aiko Tanaka, Tomonori Yamashita, Yukiko Koyama, Akinori Uchiyama, Yuji Fujino

**Affiliations:** 1,4,5 Department of Anesthesiology and Intensive Care, Osaka University Graduate School of Medicine, 2-15 Yamadaoka, Suita, Osaka, Japan; 2 Department of Anesthesiology, Osaka Women's and Children's Hospital, 840 Murodo-cho, Izumi, Osaka, Japan; 3 Department of Anesthesiology, Osaka International Cancer Institute 3-1-69 Otemae, Chuo-ku, Osaka, Japan

## Abstract

**How to cite this article:** Tanaka A, Yamashita T, Koyama Y, Uchiyama A, Fujino Y. Predictors of Successful Extubation in Reintubated Patients: The Impact of Fluid Balance during the 24 Hours Prior to Extubation. Indian J Crit Care Med 2019;23(7):344–345.

Sir,

The liberation from mechanical ventilation is a critical moment for patient outcome. The spontaneous breathing trial (SBT) and rapid shallow breathing index (RSBI) are commonly used as standard criteria.^1^ Unfortunately, re-intubation is still required at a rate of 10–20% in extubated patients.^2^ Multiple risk factors of re-intubation have been investigated, such as age, underlying pneumonia or sepsis.^3,4^ Fluid balance has been reported as a crucial physiological risk factor for extubation failure,^5^ however, its impact remains uncertain.

We present the findings of a retrospective study in successfully extubated patients after re-intubation between April, 2014 and March, 2017. The aim was to describe the difference in fluid and respiratory status between failed and successful extubations, and evaluate fluid balance as a predictor of successful extubation. The study was approved by the Institutional Review Board for Clinical Research. We recorded respiratory data of SBTs, body weight (BW) on the day of extubation, fluid balance during the 24 hours prior to extubation (24h-FB) and cumulative fluid balance (CFB). Re-intubation was defined as a need for intubation within 48 hours after the planned extubation. Wilcoxon rank sum test and Spearman's rank correlation coefficient were performed.

A total of 1837 mechanically ventilated patients were admitted to the intensive care unit during the study period, 20 post-operative patients met the inclusion criteria. The median age of the patients was 68.5 (interquartile range [IQR] 57.5, 74.5) years old, and the median weight was 58.3 (IQR 54.9, 62.7) kg. Acute Physiology and Chronic Health Evaluation (APACHE) III score was 54 (IQR 43.5, 64.8) and 15 (75%) were female patients. Cardiovascular surgery was performed for 12 (60%) patients and second extubations were excuted 89.1 (IQR 53.7, 118.0) hours after the first extubations. All extubations were performed immediately after successful SBTs using a low level of pressure support and confirmation of stable RSBIs (less than 105). There were no significant differences in PaO_2_/FiO_2_ or sequential organ failure assessment score between the first failed and the second successful extubation (Table 1). The 24h-FB and BW were significantly lower in the successful extubations although a statistically significant difference in CFB was not detected. A significant correlation was found between the changes in BW and changes in CFB (Spearman's rho: 0.57, *p* = 0.01). The study patients received fewer fluid administrarion during the 24 hours prior to the second successful extubation.

**Table 1:** Fluid and respiratory status during two series of mechanical ventilation

	*First extubation*	*Second extubation*	*p*
Fluid status before extubation			
Body weight, kg	60.6 (54.8, 65.4)	58.6 (53.9, 63.5)	0.03
24h-FB, mL	846 (–784, 208)	−357 (–943, 719)	0.02
CFB, mL	−160 (–624, 186)	695 (–911, 1691)	0.31
Fluid management during the 24 hours prior to extubation			
Fluid administration, mL/h	151 (123, 192)	111 (99, 124)	0.01
Urine output, mL/h	135 (106, 212)	116 (84, 159)	0.11
Furosemide administration, mg/h	0 (0, 2.8)	2 (0, 3.2)	0.09
Respiratory data			
RSBI, breath/min/L	32.0 (22.4, 34.8)	37.1 (29.0, 44.9)	0.04
PaO_2_/FiO_2_, mm Hg	309 (259, 370)	340 (258, 374)	0.65
SOFA score	7 (5.3, 8.0)	7.5 (6.0, 8.8)	0.51

Values are median (interquartile range)

24h-FB, fluid balance during the 24 hours prior to extubation; CFB, cumulative fluid balance; RSBI, rapid shallow breathing index; SOFA, sequential organ failure assessment

A representative cohort study of 900 patients reported that a positive 24h-FB was an essential predictor of re-extubation.^2^ However, a predictive effect of CFB has not been demonstrated in critically ill patients due to inaccuracy in recording daily fluid therapy and insensible fluid loss, which may increase over time.^5^ Although fluid balance over a shorter duration prior to extubation has been reported to be a more accurate predictor,^4,5^ there is lack of agreement regarding the duration considered significant in current clinical practice.

This cohort study clearly identified an advantage of 24h-FB; however, there was no significant difference in CFB between failed and successful extubations. Furthermore, there was significant correlation between the change in BW and change in CFB from the first to the second extubation attempts, supporting the validity of measuring fluid balance across this short duration.
